# Pre-training Model Based on Parallel Cross-Modality Fusion Layer

**DOI:** 10.1371/journal.pone.0260784

**Published:** 2022-02-03

**Authors:** Xuewei Li, Dezhi Han, Chin-Chen Chang

**Affiliations:** 1 College of Information Engineering, Shanghai Maritime University, Shanghai, China; 2 Department of Information Engineering and Computer Science, Feng Chia University, Taichung, Taiwan; Fuzhou University, CHINA

## Abstract

Visual Question Answering (VQA) is a learning task that combines computer vision with natural language processing. In VQA, it is important to understand the alignment between visual concepts and linguistic semantics. In this paper, we proposed a Pre-training Model Based on Parallel Cross-Modality Fusion Layer (P-PCFL) to learn the fine-grained relationship between vision and language. The P-PCFL model is composed of three Encoders: Object Encoder, Language Encoder, and Parallel Cross-Modality Fusion Encoder, with Transformer as the core. We use four different Pre-training missions, namely, Cross-Modality Mask Language Modeling, Cross-Modality Mask Region Modeling, Image-Text Matching, and Image-Text Q&A, to pre-train the P-PCFL model and improve its reasoning and universality, which help to learn the relationship between Intra-modality and Inter-modality. Experimental results on the platform of Visual Question Answering dataset VQA v2.0 show that the Pre-trained P-PCFL model has a good effect after fine-tuning the parameters. In addition, we also conduct ablation experiments and provide some results of Attention visualization to verify the effectiveness of P-PCFL model.

## Introduction

With the continuous development of computer vision technology and natural language processing technology, researchers go deeper in the Visual Question Answering (VQA) research field. In 2015, Agrawal et al. [1] proposed the task of a free-form and open Visual Question Answering system for the first time in VQA. Attention Mechanism [2, 3] is one of the mainstream technologies in VQA task. The method based on the Attention Mechanism can accurately capture the subject information of questions and images by weighting the Attention of questions or images and enhancing the interaction between vision and language. On the basis of the Co-Attention Mechanism’s successful application in the shallow layer network, Modular Co-Attention Networks (MCAN) [4] is a network that expands Co-Attention Mechanism further into the deep layer model. Multi-modality Fusion models such as MFH [5], DFAF [6], and MUREL [7] can also effectively improve the model’s reasoning ability, since VQA is a multi-modal task.

Although above-mentioned VQA models have achieved obvious results, the application of the models lacks universality. In recent years, researchers have proposed a Pre-training model based on the Transformer structure [8], which has achieved breakthrough performance improvements in the field of natural language processing and has been applied to the field of visual language. Pre-training [9] is an important technology in two aspects of computer vision and natural language, whose models can fine-tune different downstream tasks. Some representative methods, including ViLBERT [10], VLBERT [11], LXMERT [12], UNITER [13], OSCAR [14], VisualBERT [15], etc., use the Bidirectional Encoder Representations from Transformers (BERT) structure and have achieved effective results in VQA.

**Fig 1:**
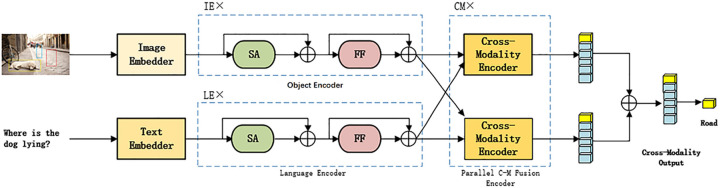
P-PCFL network framework. The model is mainly composed of three encoders, input pictures and corresponding questions, the model will return the answer to the question. For pictures of dogs, reprinted from [VQA Consortium] under a CC BY license, with permission from [VQA Consortium], original copyright [2017].

Based on the Pre-training model and the BERT structure, this paper proposes a Pre-training Model Based on Parallel Cross-Modality Fusion Layer (P-PCFL). P-PCFL is composed of three Encoders: Object Encoder, Language Encoder, and Parallel Cross-Modality Fusion Encoder, using Transformer as the core component. The overall framework of the model is shown in Fig 1. First, the input image and text pass through respective Embedders and enter the Object Encoder and Language Encoder, respectively. The Cross-Modality representation is then learned in the Cross-Modality Fusion Encoder. Finally, the predicted answer is the output. We pre-trained the model using four different Pre-training tasks, namely Cross-Modality Mask Language Modeling, Cross-Modality Mask Region Modeling, Image-Text Matching, and Image-Text Q&A, for the reason that the model can more effectively learn the Cross-Modality relationship between vision and language, as well as the fine-grained alignment between words and image regions. Multi-Modality Pre-training tasks are different from Single-Modality pre-training task, which establish a close relationship between Intra-modality and Inter-modality, thus increasing of the model’s reasoning ability.

In this paper, we fine-tuned the VQA task and tested it on the dataset VQA v2.0. to verify the validity of P-PCFL. The results show that P-PCFL is superior to other models in solving the YES/NO and counting problems. The model achieved an accuracy rate of 72.50% on Test-dev and 72.63% on Test-std. Finally, we conducted ablation studies and comparative tests to demonstrate the effectiveness of the model.

### Main contributions of this paper

- The P-PCFL model is proposed. The model is composed of three Encoders. The Parallel Cross-Modality Fusion Encoder can not only help fuse text and image information, but also establish the alignment relationship between the multi-modalities.
- Four different Multi-Modality Pre-training tasks are used to pre-train the model. The Visual Question Answering task is fine-tuned and achieves good results.
- The validity of the model is proven by ablation studies, visualization experiments, and comparative experiments.

The rest of this paper is structured as follows. The second part describes the work related to the model, the third part introduces the overall framework of P-PCFL model and the structure of each part, and the fourth part introduces Pre-training tasks and Pre-training procedures. The ablation experiment and visualization analysis are carried out in the fifth part, and finally, the last part summarizes the paper.

## Related work

### Pre-training

Pre-training is an important technology in the field of Computer Vision and Natural Language Processing. With the development of technologies such as Transfer Learning [16], Self-Supervised Learning [17, 18], and Unsupervised Learning [19], Using external data sets to conduct effective pre-training has become an important research and technical means.

On the basis of large-scale visual and text data sets, the model’s performance can be significantly improved by pre-training the basic model and migrating it to downstream tasks. In early visual dialogue methods, the visual and the language modules were pre-trained independently, which resulted in that the connection between visual data and language data was not captured effectively in the previous pre-training process. However, in recent years, researchers have found that a Pre-training model based on the Transformer structure has better performance on Natural Language Processing, and applied the model with Transformer to the field of Visual Language [20–22]. Some representative works include ViLBERT [10], VLBERT [11], LXMERT [12], UNITER [13], OSCAR [14], VisualBERT [15], etc., most of which adopt the structure of BERT. In practice, Pre-training tasks include Masked Language Modeling and Cross-modality Matching. With the help of Cross-Modality Self-Supervised Pre-training tasks on large-scale data sets, the above-mentioned methods have achieved the best performance in multiple downstream tasks, including VQA.

### Self-attention and transformer

The essence of the Attention mechanism comes from the human visual attention mechanism. When people perceive things visually, they often observe and pay attention to a specific part according to their needs. And when people find that something they want to observe often appears in a certain part of a scene, people will learn to pay attention to similar scenes in the future. The attention function can be described as a mapping from a query to a series of key-value pairs. When calculating attention, first calculate the similarity between the query and each key to obtain the weights, and then use the softmax function to normalize these weights. Finally, the weight and the corresponding value are weighted and summed to get the final attention. Self-attention is a special case of Attention. In self-attention, each unit in the sequence and all units in the sequence perform attention calculation. This calculation method can calculate the dependency between words and learn the internal structure of a sentence. In the Transformer, the Self-Attention mechanism is used to replace the entire framework built by RNN, and at the same time, a multi-head Attention mechanism is proposed (Self-Attention is done h times). The model is composed of an encoder and a decoder, and has achieved advanced results in NLP tasks, and has a faster training speed compared to other models.

### BERT in multi-modality

Since BERT was proposed, the benchmark performance of various NLP tasks has been greatly improved thanks to the powerful feature learning capabilities of Transformer. In 2019, VideoBert [23] was the first to apply BERT to a Multi-Modality model in terms of BERT’s strong learning ability. Since then, BERT has gradually been used in the field of Multi-Modality, whose application is mainly divided into two branches: single-stream model and dual-stream model.

The text information and visual information are fused in the single-stream model, while in the dual-stream model, the text information and visual information pass through two independent coding modules and then achieve the fusion of different modal information based on the Co-Attention Mechanism. VisualBERT, Unicoder-VL [24], VL-BERT, ViLT-B/32 etc., as single-stream models, all adopt the stacked Transformers’ structure to align and fuse text and image information through Transformer’s Self-Attention mechanism at the beginning of the model. The ViLT-B/32 model is a lightweight single-stream model. Although it has a faster speed, it is slightly insufficient in terms of model accuracy. ViLBERT, LXMERT, etc., as dual-stream models, do not directly integrate language and image information directly at the beginning, instead, they are first encoded by the Transformer Encoder separately and then output through a Co-Attention mechanism module. The most distinctive feature of the dual-stream model is the way visual, textual, and multi-modal information is processed in the respective encoders.

Both single-stream and dual-stream models need to be pre-trained on a large-scale data set, and then fine-tuned for the task of visual question answering [25]. In addition, these models migrate the BERT framework to the Multi-Modality field, and similarly, establish a universal feature learning model in this field.

## Model framework

The framework of P-PCFL model is shown in Fig 1. The model is mainly composed of five parts: Image Embedder, Text Embedder, Object Encode, Language Encoder, and Parallel Cross-Modality Fusion Encoder, and it accepts two inputs, namely an image and a related question. After the image and text pass through their respective Embedding layer and Encoding layer, they are then input into the Parallel Cross-Modality Fusion Encoder layer to learn the cross-modality alignment relationship between the text and image, and finally the predicted answer is output. We will describe the five components of the model in detail.

### Embedder

The Embedding layer of the model consists of an Image Embedder and a Text Embedder. The Embedding layer converts the input image and text into two feature sequences of the model, which are sent to the next layer for further processing.

**Fig 2:**
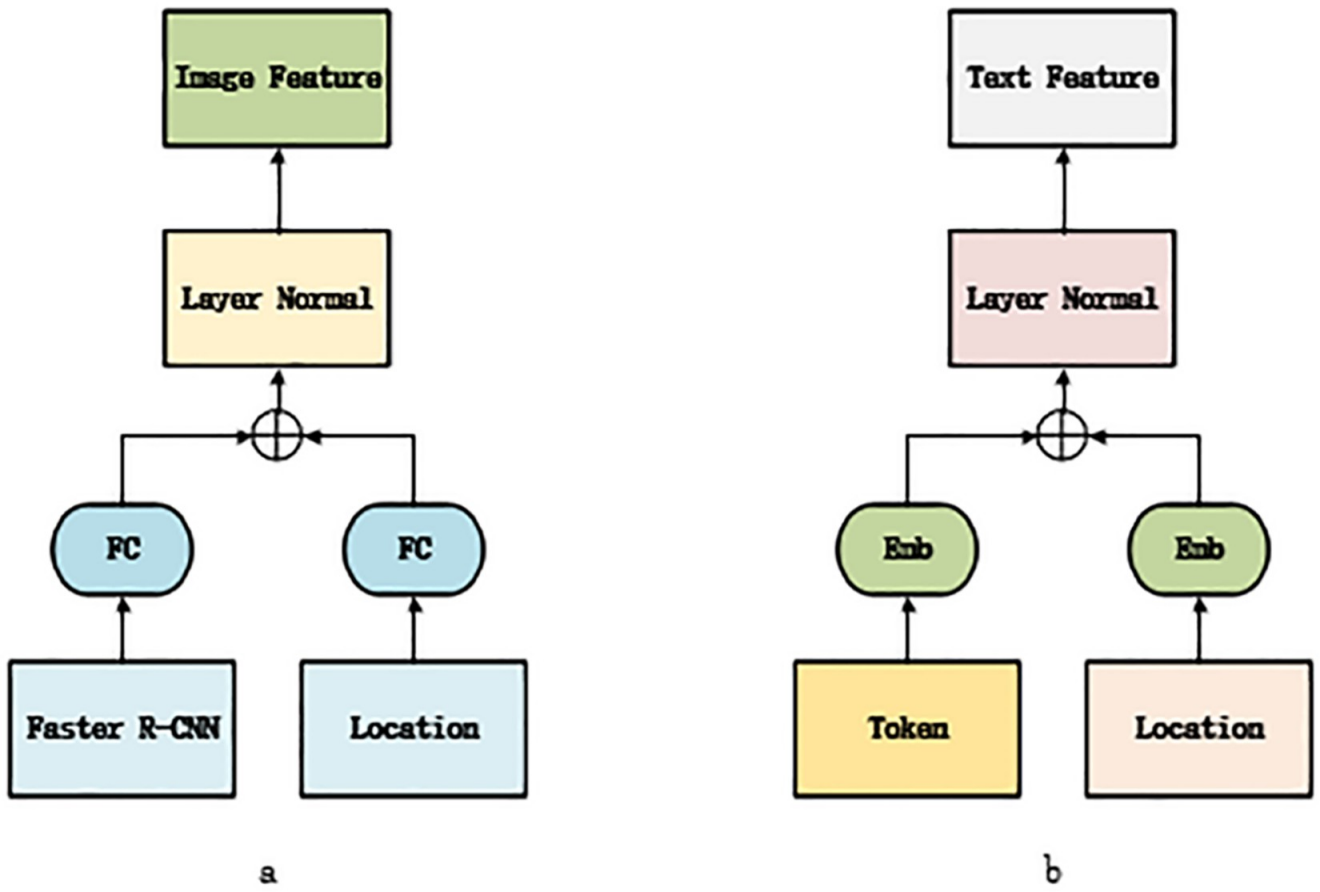
Image embedder (a) structure and text embedder (b) structure.

Image Embedder: In Image Embedder, as shown in Fig 2A, firstly, we use Faster R-CNN to extract m regions and the visual features of each region (the feature set of interest ROI, the dimension is 2048). At the same time, the location features of each area are coded. Then, we pass the visual features and location features into a public embedding space through a Fully Connected layer (FC). The outputs of the two fully connected layers are added together and then input into a Layer Normalization layer (Layer Normal) so that the final visual embedding feature of each region is obtained. The specific process is as follows:

$$\hat{VC}=FullyConnected(VC)$$ (1)

$$\hat{PC}=FullyConnected(PC)$$ (2)

$$VF=LayerNorm(\hat{VC}+\hat{PC})$$ (3)

Among them, VC is the feature set of interest ROI, PC is the location feature of each region, and VF is the final visual embedding feature representation.

Text Embedder: In Text Embedder, as shown in Fig 2B, the text of the question is transformed into a word vector of length n. Similar to the final representation of the image, the final representation of the Text Embedding feature comes from a Layer Normalization layer after the addition of its word embedding and position embedding. The specific process is as follows:

$$\hat{w_{i}}=WordEmbedding(w_{i})$$ (4)

$$\hat{o_{i}}=IndexEmbedding(o_{i})$$ (5)

$$f_{i}=LayerNorm(\hat{w_{i}}+\hat{o_{i}})$$ (6)

Among them, w_*i*_ is the word vector representation, o_*i*_ is the absolute position index of the word in the sentence, and *f*_*i*_ is the final text embedding feature representation.

### Language Encoder

Language Encoder is a single-modality encoder connected after the Text Embedder. It consists of a Self-Attention Sub-layer (SA) and a Feed-Forward Sub-layer (FF) with two Fully Connected layers, each layer of which is shown in the upper left dashed box of Fig 1. After each sub-layer, we added Residual Connection and Layer Normalization. The output *f*_*i*_ of the Text Embedder is passed to the Language Encoder and the specific process is as follows. The output of the encoder (${\hat{Q}}^{k}$)’s last layer is used as the input of the Parallel Cross-Modality Fusion Encoder layer. The Language Encoder uses the LE layer and we will determine the value of LE in the experimental part.


$$Q^{k}=SelfAttention\;(f_{1}^{k-1},\ldots ,f_{n}^{k-1})$$ (7)

$${\hat{Q}}^{k}=FeedForward\;(Q^{k})$$ (8)

### Object Encoder

The Object Encoder also consists of a Self-Attention Sub-layer and a Feed-Forward Sub-layer, which is similar in structure to the Language Encoder. As shown in the dashed box at the bottom left of Fig 1, the input of the Object Encoder is the final output of the Image Embedder, represented by *VF*. Transformer is used for both language coding and image coding in Object Encoder, while it is only used in language coding in BERT. The specific process is as follows. The output of the encoder’s last layer is used as the input of the Parallel Cross-Modality Fusion Encoder layer. The Object Encoder uses the *IE* layer and the value of *IE* is determined in the experimental part.


$${\vec{VF}}^{k}=SelfAttention\;({vf}_{1}^{k-1},\ldots ,{vf}_{m}^{k-1})$$ (9)

$${\hat{VF}}^{k}=FeedForword\;({\vec{VF}}^{k})$$ (10)

### Parallel Cross-Modality Fusion Encoder

**Fig 3:**
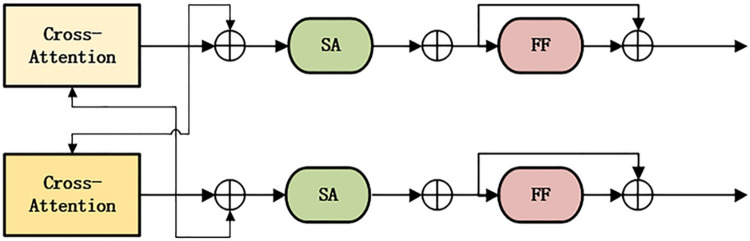
Single Cross-Modality Fusion Encoder structure.

The Parallel Cross-Modality Fusion Encoder is composed of two single Cross-Modality Fusion encoders, as shown in the dashed box on the right side of Fig 1. Each Cross-Modality module is composed of a Bidirectional Cross-Modality Attention sub-layer, two Self-Attention sub-layers, and two Feed-Forward sub-layers, as shown in Fig 3. Similar to the single-modality encoder, we add Residual Connection and Layer Normalization after each sub-layer. The Bidirectional Cross-Modality Attention sub-layer contains two unidirectional Cross-Attention Sub-layers: the vision-to-text Attention Sub-layer and the text-to-vision Attention Sub-layer, enabling the information between the two modalities to be exchanged and the entities between the two modalities to be aligned to learn the Cross-Modality joint representation better, as shown below. The Parallel Cross-Modality Fusion Encoder uses the *CM* layer and the value of *CM* is determined in the experimental part.


$${\tilde{Q}}_{i}^{k}={CMAttention}_{L\to R}({\hat{Q}}_{i}^{k-1},\{{\hat{VF}}_{1}^{k-1},\ldots ,{\hat{VF}}_{m}^{k-1}\})$$ (11)

$${\tilde{VF}}_{j}^{k}={CMAttention}_{R\to L}({\hat{VF}}_{j}^{k-1},\{{\hat{Q}}_{1}^{k-1},\ldots ,{\hat{Q}}_{n}^{k-1}\})$$ (12)

Among them, the input of the kth layer is the output of the k-1th layer, *L*→*R* is the text-to-vision Cross-modality Attention layer, and *R*→*L* is the vision-to-text Cross-modality Attention layer.

The internal modal connection can be further established by connecting the Self-Attention layer behind the Cross-modality Attention layer. The specific process is as follows:

$${\bar{Q}}_{i}^{k}={SelfAttention}_{L\to L}({\tilde{Q}}_{i}^{k},\{{\tilde{Q}}_{1}^{k},\ldots ,{\tilde{Q}}_{n}^{k}\})$$ (13)

$${\bar{VF}}_{j}^{k}={SelfAttention}_{R\to R}({\tilde{VF}}_{j}^{k},\{{\tilde{VF}}_{1}^{k},\ldots ,{\tilde{VF}}_{m}^{k}\})$$ (14)

Among them, the input of the *k*-layer is the output of the Cross-modality Attention layer’s *k*-layer, *L→L* is the Self-Attention layer from text to text, and *R→R* is the Self-Attention layer from vision to vision.

Two single Cross-Modality Fusion Encoders will output a Cross-Modality Vector respectively. A special marker [CLS] is set in front of each question during Text Embedding. We use the feature vector in the language feature sequence that corresponds to the special marker (shown in yellow) as the Cross-Modality output, that is, the output of the single Cross-Modality Fusion encoder. The two Cross-Modality outputs are added and used as the final output of the P-PCFL model. The specific process is as follows. It is found that the Parallel Cross-Modality Fusion Encoder can learn much more fine-grained relationships than the single multi-modality fusion encoder, so as to improve the model’s reasoning ability.


$${CMO}_{i}=FeedForward({\bar{Q}}_{i})+FeedForward({\bar{VF}}_{i})$$ (15)

$$CMO={CMO}_{1}+{CMO}_{2}$$ (16)

## Pre-training

Four different Multi-Modality Pre-training tasks are used to pre-training the model on large-scale data sets to help improve P-PCFL reasoning ability and understand the alignment relationship between vision and text.

### Cross-Modality Mask Language Modeling

In the Cross-Modality Mask Language Modeling task, we use a setting similar to that in the BERT model to mask the text words with a probability of 15%and replace them with a special mark [MASK]. The modeling predicts the words to be replaced by minimizing the negative logarithm likelihood and observations of words around [MASK] and m image regions.

**Fig 4:**
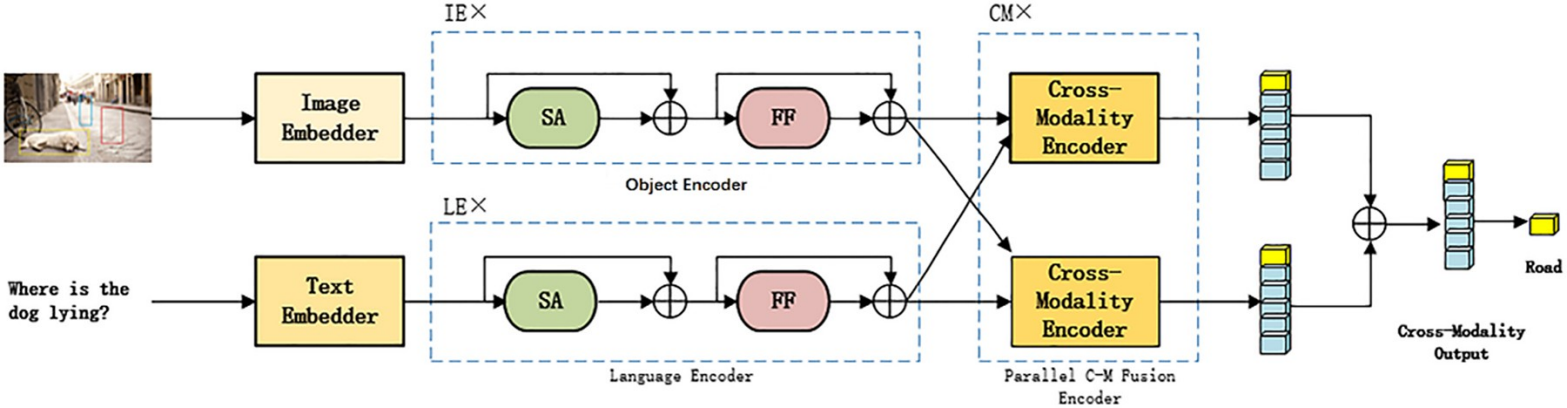
P-PCFL pre-training tasks structure. On the right side of the figure we show the results of five pre-training tasks For pictures of dogs, reprinted from [VQA Consortium] under a CC BY license, with permission from [VQA Consortium], original copyright 2017].

As shown in the lower part of Fig 4, the [MASK] tag replaces the “is” and “dog” words in the question. The model combines the context word sequence with the image region characteristics to predict the masked word successfully. The Cross-Modality Masking Language Modeling task is a Multi-Modality Pre-training task, making it helpful to better learn the relationship between various modalities and improve the reasoning ability of the model than traditional language masking task.

### Cross-Modality Mask Region Modeling

Similar to the Cross-Modality Mask Language Modeling task, the Cross-Modality Mask Region Modeling randomly masks the image region with a probability of 15%and covers the ROI feature with 0). The model is required to predict these masked objects, which means that reconstructing certain areas of the fixed image, as shown in the top branch of the Fig 4. We use the surrounding image and text information to make predictions for the masked image area. In the Cross-Modality Mask Language Modeling pre-training task, we complete the pre-training of vision with two subtasks.

(1) Masked ROI-Feature Regression

Each region has a high-dimensional feature vector. We use L2 loss [26] regression analysis to make the two features as similar as possible because the model is required to predict the high-dimensional feature vectors and make the predicted feature vector close to the feature vector of the masked region. As shown in Fig 4, through the Masked ROI-Feature Regression task, we finally get the ROI Feature Region.

(2) Masked Label Classification

The model is required to learn to predict the semantic class of the object (i.e. the label of the object) in each masked area. The Pre-training image does not provide object level annotation due to the lack of basic real label, so we use Faster R-CNN output detection tags to pre-train by minimizing the loss. As shown in Fig 4, through the Masked Label Classification task, the label of dog is predicted.

### Image-Text Matching

In the Image-Text Matching task, the input of the model is a sentence and a set of image regions, and the output is a value between (0,1) indicating whether the sentence is matched with the image region. As shown in Fig 4, we immediately replace each sentence in the task with an unmatched sentence with a probability of 50%. It can predict whether the image and sentence match by introducing a special flag [CLS] to represent the fusion of text and image and extracting the [CLS] tag to send into the FC layer and the sigmoid function.

### Image-Text Q&A

Image-Text Q&A is related to the Image-Text Matching task. In the Image-Text Matching task, in which the model can predict the answer to the image question when the question is matched with the image. In addition, we used multiple Q&A data sets to expand the pre-trained data set. As shown in Fig 4, when the result of Image-Text Matching task is “YES”, we can get the answer to the input question through Image-Text Q&A.

### Pre-training data set

Pre-training data set consists of five components: MS COCO data set [27], Visual Genome data set [28], GQA data set [29], VQA v2.0 data set, and VG-QA data set [30]. We collected the strictly separated data from the train set and dev set in each data set, and excluded all validation sets and test sets that appeared in downstream tasks to avoid overlap between pre-training and testing. As shown in the Table 1, a large image and language data set is formed with a total of 180,000 different images, and 9.18 million images and sentences are aligned on different images.

**Table 1:** Pre-training data set.

Image		Questions				
	MS COCO	VG	VQA v2.0	GQA	VG-QA	All
180K	617K	5.39M	658K	1.07M	1.44M	9.18M

### Pre-training settings

We pre-trained the P-PCFL on a large-scale data set. Each data set is divided to ensure that all the test images do not cover any pre-training images and fine-tuning images. Since MS-COCO is a relatively large validation set, a set of 5k images are extracted from the MS-COCO validation set to form a mini validation set and other images in the validation set are used as the training set. We pre-trained all encoder parameters and initialize the model parameters randomly or set them to zero. Multiple LOSS is involved and added with the same weight in P-PCFL model because the model needs multiple Pre-training tasks to learn the relationship between modalities. The Adam Optimizer [31] and a Linear Decay Learning Rate with a peak value of 1e-4 are used to plan, and the training task is divided into 20 stages with a batch size of 256. For the pre-training task of the image problem, we only trained the past 10 periods because of its faster converges and smaller learning speed.

### Fine tuning

In this paper, we fine-tune the P-PCFL model on the data set VQA v2.0 in the VQA task. The data in Train and Val is used to fine-tune the model and test our model on the Test-dev and Test-std of VQA v2.0.

## Experiment

In this section, the selected experimental data set and the setting of model parameters is introduced and the experimental results will be displayed. The effectiveness of our model is analyzed with ablation experiments and the validity of the model is further analyzed by Attention Visualization. Furthermore, a contrast experiment is conducted to compare our P-PCFL model with the existing VQA model to prove the improvement in accuracy of our model.

### Experimental data set

We use the VQA v2.0 data set to evaluate the model For the Visual Question Answering task. VQA V2.0 consists of a train set, a validation set and a test set. The train set contains 82783 images, 443757 questions and 4437570 ansewers. Validation set contains 40504 images, 2143534 questions, 2143540 answers; The test set contains 81434 images, 447793 questions. And all questions are divided into three categories: YES/NO, numbers, and others. Compared with the VQA v1.0 data set, the VQA v2.0 data set mainly solves the problem of unbalanced answers. The VQA v2.0 data set guarantees that two different pictures with the same question have different answers, which means that VQA system have to capture and use image features to generate correct answers.

### Experimental settings and model parameters

In the Embedding layer, the text input is converted into a word vector by the Word-piece Tokenizer in BERT, while the image input is used to detect 36 objects with the Faster R-CNN object detection model. In the Encoding layer, we set the layer number LE of the Language Encoder, the layer number IE of the Object Encoder, and the layer number CM of the Parallel Cross-Modality Fusion Encoder to 9, 4, and 5, respectively. The size of the hidden layer in the Encoder is set to 768, the latent dimension d in the Multi-head Attention of each Encoder is set to 512, the number of heads h is set to 8, and the latent dimension of each head is 64. We set the Basic Learning Rate of the P-PCFL model to 5e-5, the batch size to 32, and fine-tune the model according to the parameters of the pre-trained 4 epochs. To prevent overfitting, the dropout in each Fully Connected Layer is set to 0.1.

### Ablation experiment

In order to verify the effectiveness of the P-PCLF model, we conducted ablation experiments on the VQA v2.0 data set.

Number of **iterations.** As shown in Table 2, the number of iterations has effect on the accuracy of the P-PCLF model. We set the number of iterations N as 3,4,5, and 6, respectively. When the number of iterations is 4, the highest accuracy is obtained in the VQA task. Therefore, optimal model sets the number of iterations to 4.

**Table 2:** Effect of iteration number on model accuracy.

The number of Epoch	Accuracy
Epoch = 3	71.06
**Epoch = 4**	**72.50**
Epoch = 5	72.32
Epoch = 6	72.15

The number of layers of the **p**arallel **c**ross-**m**odality **f**usion **e**ncoder. It is ensured that the values of the Language Encoder’s layer number LE and the Object Encoder’s layer number IE remain unchanged to know the impact of the number of the Parallel Cross-Modality Fusion Encoder’s layers on the accuracy of the model. According to multiple experiments, the accuracy of the model is the highest when LE and IE are set to 9 and 4 respectively. The CM value is set to 3, 4, 5, 6, and 7. As shown in Table 3, the accuracy of the model is the highest when CM = 5.

**Table 3:** Effect of the number of CM layers on model accuracy.

The Layer of CM	Accuracy
LE = 9、IE = 4、CM = 3	70.09
LE = 9、IE = 4、CM = 4	71.12
**LE = 9、IE = 4、CM = 5**	**72.50**
LE = 9、IE = 4、CM = 6	71.35
LE = 9、IE = 4、CM = 7	71.19

### Visualization

Visualizing the Attention can illustrate the correspondent relationship more clearly between the image and the text, so as to further analyze the effectiveness of the model. For the P-PCFL model, we visualized the Attention of the last layer in the Parallel Cross-Modality Fusion Encoder.

**Fig 5:**
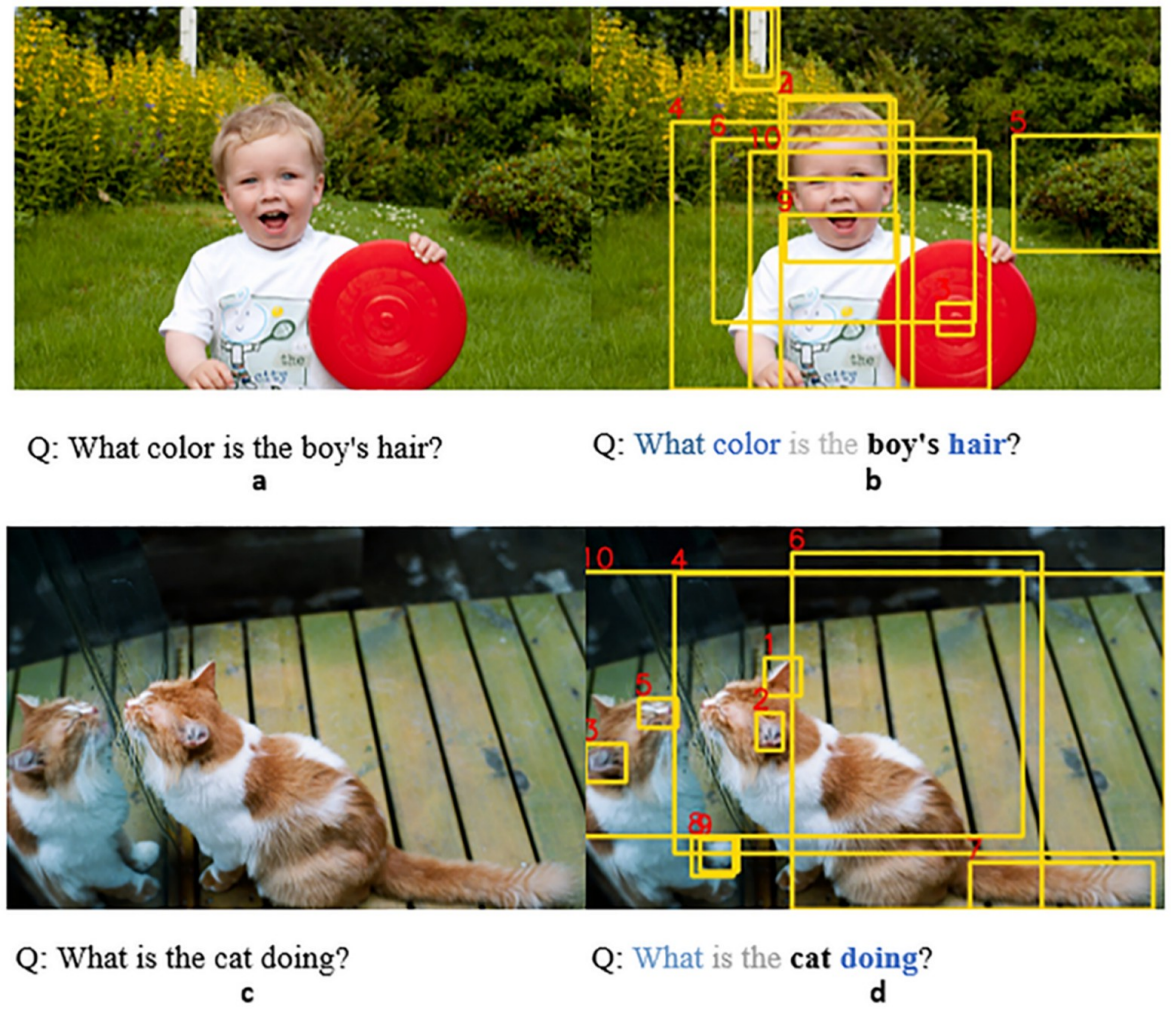
Attention visualization. A and B are in a group, c and D are in a group For pictures of boy and cat, reprinted from [VQA Consortium] under a CC BY license, with permission from [VQA Consortium], original copyright [2017].

As shown in Fig 5, each set of examples consists of two parts: the question and the image. The right side shows the Attention learned by the model. For each image, we selected the top 10 areas with the highest Attention scores in the image and ranked with the boxes. For each text of question, we bolded the keywords and highlighted words with larger Attention power weights. According to the results of visualization, the Attention of the text is mainly focused on the entity words and question words, and thege Attention area of the image is selected based on the attention of the text. In this way, the model establishes the alignment between the image and the text. In a and b of Fig 5, according to the input problem, the Attention of the text is focused on “boy”, “hari” and “color”. The Cross-Modality Fusion model combines the attention of the text to locate the position in the picture, so image’s Attention is focused on yellow box area, which is the same in Fig 5C and 5D. According to the visualized results of Attention, it shows that the P-PCFL model can learn the relationship between multiple modalities, and this relationship is one-to-one correspondence. At the same time, based on the visualized results, the learning ability of the model can be analyzed more intuitively, which provides a benchmark for improving the model.

### Comparative experiment

In this part, we compare our P-PCFL model with existing visual question answering models. As shown in Table 4, all models are performed under the same experimental settings. The DFAF model focuses on the transmission of information between Intra-modality and Inter-modality, and proposes a Multi-Modality fusion method that dynamically transmits information in visual and language modalities; the MCAN model sets two attention modules in series to form a collaborative attention module. And multiple MACs are connected in series to form a deep attention network structure. MUAN is similar to MCAN, but the difference lies in the reference to the Gated Self-Attention mechanism. Because these three models have not been pre-trained, although they have achieved good results, their accuracy is low compared to other models. Therefore, it is proved that it is necessary for the P-PCFL model to have pre-training tasks.

**Table 4:** Comparison of P-PCFL model and other models on VQA v2.0.

Model	Test-dev	Test-std
DFAF	70.22	70.34
MCAN	70.63	70.90
MUAN	70.82	71.10
VisualBERT	70.80	71.00
VL-BERT	71.79	72.22
ViLT-B/32 [32]	70.34	-
ViLBERT	70.55	70.92
LXMERT	72.42	72.54
**P-PCFL**	**72.50**	**72.63**

The Visual-BERT model and the VL-BERT model are single stream models. Visual-BERT uses two pre-training tasks, the first of which has the same language mask as BERT and the second is Sentence-Image Prediction. And VL-BERT has three pre-training tasks: Language Mask, Image Label Classification, and Image Language Matching tasks. ViLT-B/32 is also a single stream model. ViLT-B/32 uses patch projection to encode pictures, and uses image language matching tasks and language mask tasks for pre-training. Compared with the three models, the P-PCFL model increased by 2.42%, 0.71% and 2.16% on Test-dev, and increased by 1.63% and 0.41% on Test-std, respectively. VL-BERT, as a dual-stream structure, encodes visual input and text input through their respective encoders, and then enters a common Attention module to fuse information between different modalitiesby using masked Multi-modal Modeling pre-training tasks and Language Image Matching pre-training task. LXMERT is similar to VL-BERT in that it is a dual-stream structure that uses five pre-training tasks: Language Mask task, Image Mask task (in this task, it is divided into two different mask tasks), Language Image Matching task, and Image Question and Answer task. Compared with two three models, our Test-dev has increased by 1.95% and 0.08%, and the Test-std has increased by 1.71% and 0.09%, respectively.

The reason for higher accuracy of the P-PCFL model is that our model is pre-trained through four multi-modality pre-training tasks. In addition, a Parallel Cross-Modality Fusion Encoder is used, and the output of the cross-modality encoder is fused during the output process to obtain more Cross-Modality information and improve the learning ability of the model effectively. In short, compared with the existing visual question answering models, our model has achieved an improvement in accuracy and effectiveness.

## Conclusion

This paper proposes a Pre-training Model Based on the P-PCFL to learn the relationship between Multi-Modalities. Three Encoders: Object Encoder, Language Encoder, and Parallel Cross-Modality Fusion Encoder are designed, and four different Pre-training tasks are used to pre-train the model. P-PCFL is a dual-stream model that encodes text and image in separate encoding layers, and then realizes semantic alignment and fusion of text and image through a Parallel Cross-Modality Fusion Encoder. Fine-tune the VQA tasks and evaluate the model on the data set VQA v2.0. Compared with the existing models, our model has achieved effective results in accuracy. Our future work includes designing more effective Pre-training tasks, researching more effective Multi-Modality fusion methods, and applying visual question answering to a wider range of scenarios.
